# THE COGNITIVE DECLINE–MORTALITY ASSOCIATION IS MODIFIED BY LIVING ALONE AND SOCIAL NETWORK: A PARADOX OF ISOLATION

**DOI:** 10.1093/geroni/igad104.2385

**Published:** 2023-12-21

**Authors:** Hiroshi Murayama, Mika Sugiyama, Hiroki Inagaki, Chiaki Ura, Fumiko Miyamae, Ayako Edahiro, Tsuyoshi Okamura, Shuichi Awata

**Affiliations:** Tokyo Metropolitan Institute of Gerontology, Tokyo, Tokyo, Japan; Tokyo Metropolitan Institute of Gerontology, Tokyo, Tokyo, Japan; Tokyo Metropolitan Institute of Gerontology, Tokyo, Tokyo, Japan; Tokyo Metropolitan Institute of Gerontology, Tokyo, Tokyo, Japan; Tokyo Metropolitan Institute of Gerontology, Tokyo, Tokyo, Japan; Tokyo Metropolitan Institute of Gerontology, Tokyo, Tokyo, Japan; Tokyo Metropolitan Institute of Gerontology, Tokyo, Tokyo, Japan; Tokyo Metropolitan Institute of Gerontology, Tokyo, Tokyo, Japan

## Abstract

Cognitive decline (CD) is a well-known mortality risk. To date, modifiers of this relationship have been insufficiently investigated. This study explored the factors modifying the association between CD and all-cause mortality among older Japanese people. A baseline questionnaire was completed with all 132,005 residents, aged ≥65 years, without long-term care insurance certification in Adachi Ward of the Tokyo Metropolitan area in July 2015 (valid response: 75,358). The final analytic sample was 74,872, excluding 486 who reported a history of dementia (mean age = 73.7 ± 6.0 years; 44.9% males). We assessed CD using a self-administered dementia checklist that was validated via the Clinical Dementia Rating Scale. The survival analysis (average follow-up of 1,657 days) showed that CD was associated with a higher mortality risk (hazard ratio [95% confidence interval] = 1.40 [1.28–1.53]). We examined the interaction between socio-demographics, health behaviors/conditions, and CD on mortality. Only household composition and social network (SN; frequency of contact with others) demonstrated significant associations with CD. The stratified analyses showed that the CD-mortality association was stronger among those with a small SN (1.62 [1.40–1.87]) than a large SN (1.26 [1.12–1.41]). Conversely, the association was weaker in living-alone individuals (1.11 [0.90–1.38]) than in cohabiting individuals (1.46 [1.33–1.61]). Although living alone and having a small SN represented “isolated” status, their modifying effects on the CD-mortality relationship were the exact opposite. Our findings highlight that the isolation type needs to be considered when implementing dementia prevention and support for older people with CD/dementia.

